# Determining Validity of Critical Power Estimated Using a Three-Minute All-Out Test in Hot Environments

**DOI:** 10.3390/ijerph18179193

**Published:** 2021-08-31

**Authors:** Yu-Hsuan Kuo, Ching-Feng Cheng, Yu-Chi Kuo

**Affiliations:** 1 Department of Physical Education, Chinese Culture University, Taipei 11114, Taiwan; gyx2@ulive.pccu.edu.tw; 2 Department of Athletic Performance, National Taiwan Normal University, Taipei 11677, Taiwan; 3 Sports Performance Lab., National Taiwan Normal University, Taipei 11677, Taiwan; 4 Department of Exercise and Health Science, National Taipei University of Nursing and Health Sciences, Taipei 11219, Taiwan; yuchi@ntunhs.edu.tw

**Keywords:** critical threshold, exercise intensity, fitness test, heat

## Abstract

The aim of this study was to investigate the effects of heat on the validity of end-test power (EP) derived from a 3-min all-out test (3MT), which is considered as an alternative method for determining the conventional critical power. Twelve male cyclists were required to perform incremental exercise tests (IET) and 3MTs in both high temperature (HT; 35 °C) and thermoneutral temperature (NT; 22 °C) environments. Maximal oxygen uptake (VO_2max_), and first and second ventilatory thresholds (VT_1_ and VT_2_, respectively) against the power output (*w*VO_2max_, *w*VT_1_, and *w*VT_2_) were measured during IETs. EP was recorded during the 3MTs. A significant correlation was observed between *w*VT_2_ and EP under NT (*r* = 0.674, *p* < 0.05) and under HT (*r* = 0.672, *p* < 0.05). However, *w*VO_2max_, *w*VT_1_, *w*VT_2_, and EP were significantly higher in NT than in HT (*p* < 0.05). In conclusion, although the physiological stress induced by HT might impair exercise performance, the EP derived from 3MT can validly estimate *w*VT_2_ under HT conditions.

## 1. Introduction

Internationally, numerous major sports events, such as the Summer Olympic Games, world cups, and world championships are held in hot summer months. Athletes in a high temperature (HT) environment must cope with external heat stress, which increases their level of fatigue and expedites exhaustion. Therefore, determining how athletes can train and compete effectively in HT environments is crucial. Acute heat stress impairs aerobic capacity and endurance performance, and elevated cardiovascular strain has long been considered as an important contributor [1,2,3,4,5,6,7].

Theoretically, critical power (CP) or critical velocity (CV) represents the upper limit of work rate or running speed that can be maintained at a metabolic steady state, and demarcates the heavy from severe exercise intensity domains [8]. Studies have used a single 3-min all-out test (3MT) as an alternative approach for determining CP, which is referred to as end-test power (EP) [9]. Previous studies have indicated that 3MT has a good test–retest reliability [10,11] and is a valid protocol for estimation of CP or CV [12,13].

Previous studies have also reported that the EP measured in temperature-controlled laboratories (21–25 °C) is correlated well with the second ventilatory threshold (VT_2_) [14,15]. However, Kuo et al. [16] reported male runners performing a 3-min all-out running test (3MRT) in a high-outdoor-temperature environment (approximately 34.5 °C). The results showed that CV had mid- to high-test–retest reliability; furthermore, the CV tested under HT was lower than the running velocity at VT_2_ under thermoneutral temperature (NT, approximately 22 °C). That is, conducting a 3MRT under HT may underestimate an individual’s CV intensity compared with a test under NT. Interestingly, whether EP measured under HT can be applied in HT environment to demarcate the heavy from severe exercise intensity domains remains unclear.

Therefore, the purpose of this study was to investigate the validity of EP derived from 3MT under HT environments by testing the correlation between EP and VT_2_ in HT. This study also examined the acute effects of HT on the physiological responses and exercise performance during 3MT and incremental exercise tests (IETs).

## 2. Materials and Methods

### 2.1. Participants and Protocol

Twelve male recreational cyclists (age 26.1 ± 5.4 years; height 1.74 ± 0.06 m; weight 67.5 ± 8.7 kg) volunteered to participate in this study. The average weekly bicycle training time was more than 6 h in the 3 months prior to this experiment. All participants provided written informed consent before participating in the experiment. The study was approved by the National Taiwan Normal University’s ethics committee on human research and conformed to the principles of the Declaration of Helsinki.

### 2.2. Procedures

In this study, all participants randomly performed the IETs under HT (ambient temperature, 35.4 ± 0.8 °C; humidity, 36.2 ± 4.7%RH) and NT (ambient temperature, 22.3 ± 0.6 °C; humidity, 39.3 ± 6.4%RH) in an environmental chamber, respectively. Participants underwent IETs under NT and HT in a randomised crossover design. Following completion of the IETs, 3MTs were also performed in a randomised crossover manner. Each participant underwent four tests separated by ≥48 h. Before the tests began, participants sat for 20-min under the same temperature before the exercise tests to acclimatise the different environmental conditions [17]. In addition, the participants weighed themselves before and after the experiment to record the dehydration conditions. During the measurement process, a portable gas analysis system (Cortex Metamax 3B, Cortex Biophysik, Leipzig, Germany) and a telemetry system with a wireless chest strap (Polar S810i; Polar Electro, Inc., Oy, Kempele, Finland) were used to collect oxygen uptake (VO_2_) and heart rate (HR) data. The rating of perceived exertion (RPE) was measurement before and immediately after the exercise tests.

### 2.3. Incremental Exercise Tests

The IETs were performed on a cycle ergometer (Cyclus 2, RBM Elektronik-Automation, Leipzig, Germany) until volitional exhaustion, according to the protocol of Bailey et al. [18]. Participants were asked to maintain their self-selected cycling cadence (90 rpm, *n* = 1; 80 rpm, *n* = 11) throughout the test. Pulmonary gas exchanges were measured breath-by-breath throughout the IETs by having the participants wear a face mask (7400 Vmask series, Hans Rudolph, Kansas City, MO, USA) attached to the portable gas analysis system. Maximal effort was considered when three of the following four criteria were achieved [10]: (1) respiratory exchange ratio > 1.2; (2) heart rate > 90% of the age-predicted maximum; (3) a plateau in VO_2_ defined as no expected increase higher than 150 mL·min^−1^, despite an increase in power output; and (4) RPE > 17. After the IETs, we exported the VO_2_ data with average values of 10 and 30 s, and used them as indicators for calculating VT and the maximal oxygen uptake (VO_2max_), respectively [11].

*w*VO_2max_ was defined as the maximum power output when VO_2max_ was reached. Three experienced researchers estimated the VT_1_ and VT_2_ and their corresponding output powers (*w*VT_1_ and *w*VT_2_) using the V-slope method in a blinded fashion [19]. If the results differed, the three researchers discussed the results until a consensus was reached.

### 2.4. Three-Minute All-Out Exercise Tests

The linear mode of the ergometer was used to set the load setting during 3MT, so that participants would attain the given power output equal to *w*VT_1_ + 50% (*w*VO_2max_ − *w*VT_1_) on reaching their preferred cadence (linear factor = power/cadence squared) [20]. *w*VT_1_ and *w*VO_2max_ were calculated from IET under the respective environmental conditions. After 20-min of temperature acclimation, the participants performed warmup (~80%VT_1_, 5 min) and conducted 3 × 10 s sprints (1-min rest between each sprint). Subsequently, the participants rested off the bicycles in a sitting posture for 20 min and returned to the bicycles during the last 1 min. The test started with 3 min unloaded, and increased to 110 rpm 5 s before the test. All participants were asked to maintain the cycling cadence as much as possible during the 3MTs.

The highest average oxygen uptake value of 10 s was determined as the peak of oxygen uptake (VO_2peak_). EP was defined as the mean power in the final 30 s of the 3MT to exhaustion, and the work-done-above EP (WEP) was defined as the power‒time integral above the EP in the 3MT.

### 2.5. Blood Samples

Blood samples were collected from the left earlobe. We obtained capillary blood samples (approximately 0.3 µL) at baseline (pre-test) and 5-min after the exercise tests (post-test) to assess the blood lactate using a Lactate Pro chemistry analyser (KDK Corporation, Kyoto, Japan).

A capillary tube was used to collect the blood until it was 80% full (approximately 20 µL). Subsequently, the collected blood was placed in a high-speed centrifuge for 10-min of centrifugation at 12,000 rpm. Three researchers individually made determinations of the haematocrit using a microhaematocrit reader.

### 2.6. Statistical Analysis

Descriptive statistics were calculated and are presented as means ± standard deviations. Statistical significance was set at *p* ≤ 0.05. Student’s paired-samples *t* tests and 95% paired-samples confidence intervals of the mean differences were used to examine the effect of two environmental temperatures on the physiology parameters and power outputs in the IETs and 3MTs. The comparisons among power outputs (*w*VO_2max_, *w*VT_1_, *w*VT_2_, and EP) in NT or HT were analysed using a one-way ANOVA with repeated measures. A two-way analysis of variance with repeated measures (ANOVA) and a least-significant-difference (LSD) post hoc test were used to investigate the main effects and interactions between treatment factors (NT vs. HT) and time factors (pre- vs. post-test) for the haematocrit, blood lactate, RPE, and body weight. Pearson product moment correlation was used to examine the correlations of aerobic capacity indicators under the same environmental temperature. Cohen’s *d* of effect size (*d*) for the differences was calculated to indicate the statistical magnitude of the significance. Cohen’s *d* between 0.20 and 0.49, between 0.50 and 0.79, and for 0.80 and above indicated small, moderate, and large differences, respectively [21]. The G*Power 3.1 was used to calculate the appropriate sample size. According to the previous studies [14,15], which recruited active individuals, the minimum sample size of *n* = 6−8 is required to obtain a strong correlation (*r* = 0.85–0.91) between *w*VT_2_ and EP with 80% power and 5% alpha.

## 3. Results

**Figure 1:**
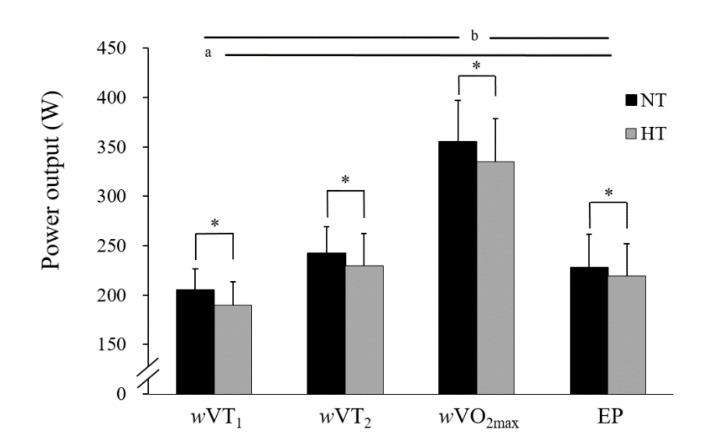
Comparison of the differences among parameters of environments and exercise intensities under thermoneutral temperature (NT) and/or high temperature (HT). * Significant difference between temperature environments (*p* < 0.05). ^a^ *w*VT_1_ was significantly different from the other intensities in NT or HT (*p* < 0.05). ^b^ *w*VO_2max_ was significantly different from the other intensities in NT or HT (*p* < 0.05).

**Figure 2:**
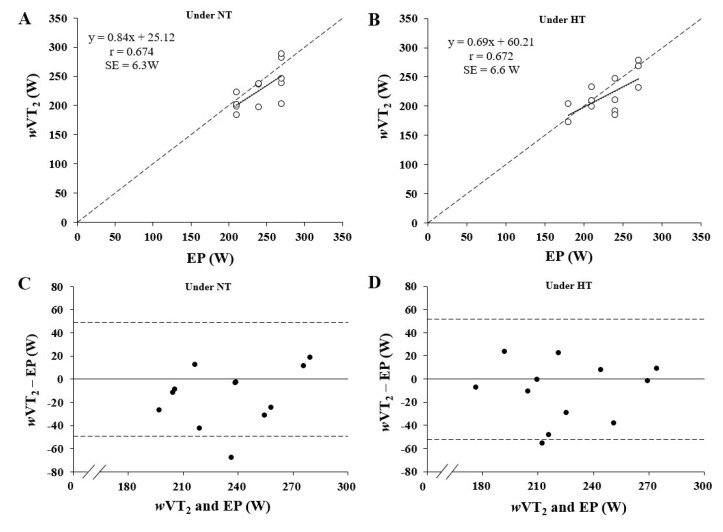
Correlation (**A**,**B**) and Bland–Altman plot (**C**,**D**) analyses for power differences between the second ventilatory threshold (VT_2_) and the end power (EP) during 3-min all-out test under thermoneutral temperature (NT) and high temperature (HT). In panels (**A**,**B**), the solid line is the best-fit linear regression and the dashed line is the line of identity. In panels (**C**,**D**), the solid line represents the mean difference between the two trials, and the dashed line represents the 95% limits of agreement.

The results indicated that there were significant differences among the *w*VO_2max_, EP, *w*VT_2_, and *w*VT_1_ (*w*VO_2_max > EP, *w*VT_2_ > *w*VT_1_, *p* < 0.05) under both environments (Figure 1). Furthermore, *w*VT_2_ showed no significant difference to EP under NT (*p* = 0.070, *d* = 1.171) and HT (*p* = 0.188, *d* = 0.816), and both parameters were significantly correlated under NT (*r* = 0.674, *p* = 0.016, *d* = 1.671) and HT (*r* = 0.672, *p* = 0.017, *d* = 1.650) (Figure 2). The VO_2max_ measured by IET and the VO_2peak_ measured by 3MT had significantly strong correlations in both NT (*r* = 0.877, *p* = 0.000, *d* = 3.650) and HT (*r* = 0.893, *p* = 0.000, *d* = 3.968).

Table 1 indicates that the VO_2max_ of the IETs under HT was significantly higher than under NT. In terms of power output, *w*VO_2max_, *w*VT_1_, and *w*VT_2_ under HT were significantly lower than those under NT. The time to exhaustion during IET under HT was significantly shorter than under NT (*p* < 0.05).

The results of the 3MTs indicated that VO_2peak_, VE, and HR_peak_ under HT were all significantly higher than those under NT (Table 2). The EP, peak power, and mean power under HT were significantly lower than those under NT; however, there was no significant difference between conditions in WEP (Table 2).

A significant interaction effect between treatment and time on body weights was found in the IET test. During the IET test, the body weights at post-test in both HT and NT were significantly lower than those at pre-test (Table 3). As shown in Table 3, the significant main effects of time were on haematocrit, blood lactate, and RPE in the IET test. The haematocrit after IET under NT was significantly higher than that under HT (*p* = 0.032, *d* = 1.011).

A significant interaction effect between treatment and time on body weights (*F* = 14.599, *p* < 0.05) was found at 3MT. Table 3 demonstrates that the body weights under NT and HT were significantly lower after 3MT compared with those before, and the body weight after 3MT under HT was significantly lower than that under NT. There were significant main effects of time on haematocrit, blood lactate, and RPE in the 3MT (Table 3).

## 4. Discussion

The primary finding of the present study is that under HT or NT conditions, the EP estimated from 3MT was similar to the *w*VT_2_ determined by IET. Therefore, the EP measured with a 3MT under HT can be used as an indicator for distinguishing high- and severe-intensity exercise domains under the same heat stress. In addition, the exercise performance during IET and 3MT was obviously impaired by the acute heat exposure.

The power output at VT_2_ can be the upper boundary of the heavy intensity domain and the lower boundary of severe intensity domain, which is equivalent to CP [12,22]. In this regard, previous studies have reported that CP/EP is highly correlated with VT_2_ [14,15], and can also be used to discriminate the heavy and severe exercise intensities [10]. However, the CV derived from the 3MRT in an outdoor hot environment only correlates well with VT_1_ that is measured in normal temperature laboratory [16]. The present study is the first study to find that the EP estimated from 3MT in HT could be used to distinguish the heavy and severe exercise intensities in HT. In addition, the EP is not interchangeable between HT and NT.

Previous studies have suggested that HT has negative effects on individuals’ exercise performance compared with NT [2,5,23]. One study demonstrated that the power outputs corresponding to maximal lactate steady-state and VT were lower at temperatures of 40 °C than at 23 °C [2]. The present study observed that *w*VO_2max_ under HT was approximately 5.6% lower than under NT. Moreover, the workload at sub-maximal exercise intensities under HT were lower than under NT. Therefore, coaches and athletes should carefully consider the declines in exercise economy and/or efficiency when competing or training in HT conditions.

Numerous studies have indicated that VO_2max_ measured under HT would be lower than that under NT [5,6,7]. However, one study suggested the possibility of increasing VO_2max_ under HT [4]. The study showed that VO_2max_ under HT (~35 °C, 69.3 mL·kg^−1^·min^−1^) was significantly higher than that under NT (~22 °C, 64.7 mL·kg^−1^·min^−1^). The total duration before IET in the study of Lafrenz et al. [4] was 35 min (20-min heat acclimation and 15-min submaximal exercise), which was close to the duration of the present study (total duration before IET was approximately 23 min; 20-min heat acclimation and 3-min warm-up). In contrast, Lafrenz et al. [4] indicated that after the 45-min warm-up, if an IET was performed, the VO_2max_ would decrease significantly (total duration before IET was 65 min; 20-min heat acclimation and 45-min submaximal exercise). If a relatively long preparation (approximately 60 min) under HT was allowed before the test, the marked skin and internal body hyperthermia would impair subsequent VO_2max_ [7]. Researchers have reported that the different preheating strategies, including active warm-up and/or passive heating, could result in different magnitudes of increases in core and skin temperatures [1]. They further indicated that the reduced VO_2max_ and physical performance might be in proportion with the increases in core and skin temperatures before performing IET [7]. Therefore, the different total time spent under heat exposure may cause different degrees of hyperthermia. Further studies are needed to clarify the effects of total time spent under heat exposure before exercise tests on the subsequent VO_2max_ and exercise performance. Nevertheless, we note that as heat exposure time increases, the VO_2max_ might be reached at a lower work rate.

This present study found that the power output performance (such as EP) during 3MTs was impaired by heat exposure. A relatively long exercise duration under HT might produce hyperthermia, which is another factor that can decrease the subsequent power performance [24]. Nybo and Nielsen [23] noted that after participants performed 50–60 min of 60%VO_2max_ cycling at 40 °C and 18 °C, the strength and electrical activity at the 60 to 120 s maximal voluntary contraction in the leg muscles under HT were significantly lower than for NT. Present study showed that all participants spent approximately 50 min (20-min heat acclimation, 7.5-min warm-up, 20-min rest, and 3-min unloaded phase) on activity preparation before the 3MTs under HT; this preparation might have decreased the power outputs, even if the exercise duration of the test was only 3 min. As mentioned above, the total time spent in heat exposure is the determinant of the impairments in exercise performance changes, not just the duration of the exercise testing.

Many underlying mechanisms, such as cardiovascular, central nervous system, peripheral, and psychological factors, may potentially contribute to premature fatigue under HT environments [25,26]. For example, the heat stress would result in an increase in skin blood flow to dissipate more heat across the skin, which reduces the venous return, thus leading to a compensation of decreased stroke volume and increased heart rate. Another explanation for the declines in performance during 3MTs in HT might be the respiratory muscle fatigue. Mitchell et al. [27] reported that heat stress could reduce the time to exhaustion during high-intensity exercise and is accompanied by greater ventilatory responses than for a cool environment. The results of this study found that the VE in HT was higher than that in NT during 3MT. Therefore, the HT might induce more intense fatigue responses in respiratory muscles than NT, and thus lower the work performance during intense exercise.

Reasons for causing an increase in haematocrit during exercise included factors such as dehydration [28] or blood viscosity [29]. The present study showed that haematocrit following IETs had a significant increase under NT than HT (NT vs. HT, +9.8% vs. +8.5%). During the approximately 1-h experimental period, participants were not allowed to drink water. This might be the reason for the increases in haematocrit. However, the body weight data before and after IETs showed that the weight loss (approximately 0.4–0.6%) was similar between HT and NT. A magnitude of decrement less than 2% of body weight has been reported to be insufficient to affect exercise performance [30]. Therefore, the dehydration condition might not be a main factor affecting the decreases in exercise performance in this study.

The results in this present study demonstrated that the EP derived from 3MT correlated well with the *w*VT_2_ under HT; however, there was a significant difference in EP values between HT and NT. The question may arise as to whether the relationship between EP and *w*VT_2_, and/or the differences on EP between HT and NT will change after heat acclimation. After heat acclimation, cardiovascular and metabolic adaptations, such as lower HR, higher sweat rate, increases in total body water and plasma volume expansion, and reduction in rate of glycogenolysis [31,32], may contribute to improvements in exercise performance under hot conditions. Lorenzo et al. [33] found that 10-day heat acclimation could delay the occurrence of lactate threshold. It seems to be expected that the EP estimated by 3MT under HT may become similar to EP under NT after proper heat acclimation. However, this current study was conducted in the early summer, and the average temperature (approximately 26–32 °C) was lower than the environmental setting of this study, but it was still considered as mild hot conditions. Therefore, further studies focused on the effects of a proper heat acclimation on EP are needed.

Like other studies, this study has certain limitations. First, only one temperature was selected for the heat exposure treatment in this study. It is remain unclear whether the same results will occur in other high-temperature environments. Second, this study was conducted using a stationary bicycle in a well-controlled environmental chamber that had been widely used in prior research, but these settings were somewhat limited in ecological validity. 3MT has been widely developed in outdoor environments [34,35]. The effects of other factors of the outdoor condition, such as wind and rain, on the 3MT remain to be clarified. Finally, our small and all-male participant sample limits the generalization of these results to other populations, especially women, particularly given the influence that menstrual cycle phase and heat tolerance capacity can have on physiological indices, such as core temperature and power output [36].

## 5. Conclusions

Under the HT environment, the EP estimated by 3MT had a similar exercise intensity to *w*VT_2_. Therefore, EP can not only validly demarcate the heavy from severe exercise intensity domains, but can also be used to assess physical fitness and prescribe training programs under HT. Nevertheless, heat exposure increases physiological stress (e.g., Hct) and decreases work performance (i.e., EP, *w*VT_1_, *w*VT_2_, and *w*VO_2max_ in HT were lower than those in NT) during exercise. For practical applications, EP should be evaluated and applied in a temperature environment where training/competition may occur in order to avoid over- or under-estimating training intensities.

## Figures and Tables

**Table 1:** Effect of different environmental temperatures on incremental exercise tests.

			95% Confidence Limits			
	NT	HT	Lower Bound	Upper Bound	*p*	d
Ambient temperature (°C)	22.5 ± 0.6	35.3 ± 0.9 *	−13.574	−11.942	0.000	14.166
Humidity (%RH)	36.3 ± 5.2	41.1 ± 7.5	−10.786	1.120	0.101	0.780
VO_2max_ (mL·kg^−1^·min^−1^)	59.3 ± 7.6	61.3 ± 8.0 *	−3.408	−0.592	0.010	1.297
VT_1_ (mL·kg^−1^·min^−1^)	37.8 ± 5.4	36.7 ± 4.9	−1.636	3.880	0.390	0.367
VT_2_ (mL·kg^−1^·min^−1^)	44.9 ± 5.8	45.0 ± 7.2	−1.809	1.582	0.886	0.073
wVO_2max_ (W)	355.0 ± 42.1	335.0 ± 44.0 *	10.615	29.385	0.001	1.929
wVT_1_ (W)	205.0 ± 21.5	190.0 ± 23.4 *	5.046	24.954	0.007	1.361
wVT_2_ (W)	242.5 ± 27.0	230.0 ± 32.2 *	2.685	22.315	0.017	1.209
Time to exhaustion (s)	879.9 ± 76.3	846.7 ± 72.8 *	24.268	42.232	0.000	3.420

Notes: NT—thermoneutral temperature; HT—high temperature; VO_2max_—maximal oxygen uptake; VT_1_—first ventilatory threshold; VT_2_—second ventilatory threshold; wVO_2max_—VO_2max_ against power output; wVT_1_—VT_1_ against power output; wVT_2_—VT_2_ against power output; *d*—effect size. * Significantly different to NT (*p* < 0.05).

**Table 2:** Effect of different environmental temperatures on 3-min all-out tests.

			95% Confidence Limits			
	NT	HT	Lower Bound	Upper Bound	*p*	d
Ambient temperature (°C)	22.1 ± 0.5	35.6 ± 0.6 *	−14.144	−12.840	0.000	18.613
Humidity (%RH)	36.1 ± 4.3	37.5 ± 4.8	−5.412	2.578	0.452	0.319
Load (N)	280.0 ± 30.9	262.5 ± 32.9 *	9.543	25.457	0.001	2.002
VO_2peak_ (mL·kg^−1^·min^−1^)	57.8 ± 8.4	61.3 ± 7.4 *	−5.161	−1.672	0.001	1.860
VE (L·min^−1^)	146.6 ± 18.3	151.7 ± 20.3 *	−8.653	−1.587	0.009	1.842
HR _peak_ (bpm)	182.1 ± 9.7	187.9 ± 12.4 *	−9.786	−1.881	0.008	1.452
EP (W)	227.9 ± 33.5	219.3 ± 33.1 *	1.990	15.332	0.016	1.163
WEP (kJ)	14.4 ± 3.0	14.6 ± 3.0	−1.641	1.145	0.702	0.160
Peak power (W)	606.3 ± 82.0	587.5 ± 86.7 *	0.633	37.033	0.044	0.944
Mean power (W)	307.9 ± 31.7	300.4 ± 34.2 *	4.256	10.635	0.000	2.384

Notes: NT—thermoneutral temperature; HT—high temperature; Load—resistance of 3MT; EP—end power; WEP—power output above EP; VO_2peak_—peak oxygen uptake; VE—ventilation; HR _peak_—peak heart rate; *d*—effect size. * Significantly different to NT (*p* < 0.05).

**Table 3:** Effect of different environmental temperatures on the physiological parameters related to exercise tests.

	IET				3MT			
	NT		HT		NT		HT	
	Pre-Test	Post-Test	Pre-Test	Post-Test	Pre-Test	Post-Test	Pre-Test	Post-Test
Body weight (kg)	67.7 ± 8.8	67.4 ± 8.8 †	67.7 ± 8.6	67.3 ± 8.6 †	67.7 ± 8.7	67.3 ± 8.7 †	67.4 ± 8.6	66.7 ± 8.5 †*
Hct (%)	49.0 ± 3.7	53.8 ± 3.6 †	48.2 ± 3.5	52.3 ± 3.4 †*	49.8 ± 3.8	54.7 ± 3.5 †	49.6 ± 2.6	54.2 ± 2.7 †
Lac (mmol·L^−1^)	0.9 ± 0.2	11.0 ± 2.4 †	1.1 ± 0.2	11.4 ± 2.4 †	0.9 ± 0.2	13.2 ± 2.4 †	1.2 ± 0.2	14.1 ± 2.6 †
RPE	6.7 ± 0.8	18.5 ± 2.8 †	6.9 ± 0.5	19.4 ± 0.7 †	6.7 ± 0.7	20.0 ± 0.0 †	6.9 ± 0.8	19.9 ± 0.3 †

Notes: NT—thermoneutral temperature; HT—high temperature; IET—incremental exercise tests; 3MT—3-min all-out tests; Hct—haematocrit; Lac—blood lactate; RPE—rating of perceived exertion. * Significantly different to NT (*p* < 0.05). † Significantly different to pre-test (*p* < 0.05).

## Data Availability

Not Applicable.
